# Integrated Health, Social, and Legal Approaches to Supporting Migrant Women Victims of Human Trafficking and Sexual Violence

**DOI:** 10.3390/healthcare13222878

**Published:** 2025-11-12

**Authors:** María del Mar Jiménez-Lasserrotte, Karim El Marbouhe El Faqyr, Maria Kinza El Amrani Escot, María José Rodas Vanegas, José Granero-Molina, José Manuel Hernández-Padilla

**Affiliations:** 1 Faculty of Health Sciences, Department of Nursing, Physiotherapy and Medicine, University of Almería, 04120 Almería, Spain; mjl095@ual.es (M.d.M.J.-L.); mariakinza99@gmail.com (M.K.E.A.E.); jgranero@ual.es (J.G.-M.);; 2 Faculty of Health Sciences, Universidad Autónoma de Chile, Santiago de Chile 7500000, Chile

**Keywords:** migrant women, human trafficking, health care, social support, legal assistance

## Abstract

**Background/Objectives:** Human trafficking is a serious violation of human rights, with migrant women being among the most affected groups. This study aimed to explore the experiences of health, legal, and social professionals involved in the care of migrant women victims of trafficking and sexual violence in southern Spain. **Methods:** A qualitative design was applied, using semi-structured interviews with 47 professionals from hospitals, NGOs, and legal institutions. **Results:** The analysis identified common challenges such as language barriers, limited resources, and the absence of standardized protocols. Health professionals highlighted the need for continuous training and culturally sensitive care; legal professionals emphasized flexibility in procedures and the importance of confidential interviews for early detection; and social workers stressed the value of coordinated action across sectors. **Conclusions:** The findings underline the need for an integrated approach that combines health, social, and legal responses in order to improve protection and support for migrant women victims of trafficking.

## 1. Introduction

In recent decades, there has been a global increase in migratory movements, with large numbers of people relocating to other places in search of a better life [1]. The main causes driving these migratory processes are poor social and labor conditions, and more specifically [2], mistreatment, gender-based violence, sexual assaults, and acts that violate women’s freedom and integrity [3].

The most recent figures published by the UNHCR in its Global Trends Report 2025 [4], reveal an unprecedented scenario in terms of forced mobility. More than 120 million people worldwide have been forced to leave their homes due to armed conflicts, persistent violence, persecution, or serious violations of fundamental rights. Within this population, one group is especially exposed to extreme vulnerability, migrant women who, after fleeing unsafe contexts, become trapped in human trafficking networks.

The phenomenon of human trafficking, far from diminishing, shows an upward trend that has intensified in recent years. The most recent Global Report on Trafficking in Persons, published by the United Nations Office on Drugs and Crime in December 2024, warns of a 25% increase in the number of identified victims compared to pre-pandemic figures [5].

In this context, and according to the aforementioned report, women and girls constitute the majority of those affected, accounting for 61% of the total in 2022, with sexual exploitation remaining the predominant form. Vulnerability is further intensified in the case of migrants [6], as irregular administrative status, the absence of family or community networks, and language barriers place them in a situation of particular defenselessness [7].

In 2024, CEAR estimated that more than 123 million people worldwide were in situations of forced displacement, a figure that illustrates the magnitude of contemporary humanitarian emergencies and the repeated violation of fundamental rights. The report highlights that current migratory flows are driven by wars, persecution, and extreme climate events, significantly affecting women who, in their flight, are exposed to additional dangers. In this context, migrant women—particularly those captured by trafficking or exploitation networks—face serious obstacles in accessing health services, social support, and legal protection. These circumstances intensify their vulnerability: while attempting to escape environments marked by violence or oppression, they are forced to travel along unsafe routes, often under conditions of sexual, labor, or psychological exploitation, without effective protection mechanisms [8].

In the Spanish context, the most recent GRETA Report on Spain [9], published in 2023, highlights both progress and persisting gaps. While GRETA welcomed the increased availability and diversity of assistance measures for female victims of sexual exploitation, it underscored that there are still no specialised shelters for women subjected to other forms of exploitation, nor for male victims of trafficking. The report urges the Spanish authorities to expand the number of specialised accommodation facilities and to enhance interinstitutional coordination for victims’ access to comprehensive healthcare, legal aid, and social support.

These observations confirm the relevance of examining integrated responses within the Andalusian context, where migrant women from sub-Saharan Africa constitute one of the most vulnerable groups.

In human trafficking for the purpose of sexual exploitation, victims are recruited through deception, aggression, physical and psychological threats, and the imposition of significant financial debts [10].

In this context, human trafficking cannot be reduced merely to an issue of irregular migration, public order, organized crime, or prostitution [11] when designing and implementing action plans to combat it; it must be addressed in all its dimensions and grounded in the framework of universal human rights [12].

In the case of trafficking of women for sexual exploitation, the commodification of these women as mere objects for profit reflects an exacerbation of capitalism [13].

We focused this investigation on women who have been trafficked because trafficking represents a distinct and particularly severe violation of human rights. It involves coercion, exploitation, and the deliberate destruction of personal autonomy [14]. Although other categories of forced migrants—such as refugees or asylum seekers—also experience extreme vulnerability, trafficked women face unique legal and institutional barriers to recognition, protection, and recovery. These include a high risk of re-victimisation, gender-based violence, and complex procedural requirements for obtaining assistance or legal status [15]. Concentrating on this group allowed the study to explore how professionals address these specific challenges and to identify service gaps that require integrated responses across the health, legal, and social sectors.

The integrated framework proposed in this study is consistent with States’ obligations under international and European human rights law. The Council of Europe Convention on Action against Trafficking in Human Beings (2005) [16] and the United Nations Protocol to Prevent, Suppress and Punish Trafficking in Persons (Palermo Protocol, 2000) [17] establish clear duties for prevention, victim protection, and access to remedies. Likewise, the European Convention on Human Rights—particularly Articles 3 and 4—and relevant case law [18] emphasise States’ positive obligations to protect individuals from inhuman or degrading treatment, slavery, servitude, and forced labour. These instruments collectively require that States guarantee coordinated, victim-centred access to healthcare, legal assistance, safe housing, and social reintegration. The findings of this study contribute to operationalising these obligations through practical recommendations for cross-sector collaboration and culturally competent service delivery.

Beyond the legal and institutional framework, this study is also grounded in an intersectional and trauma-informed perspective. This conceptual approach recognises that the experiences of trafficked migrant women are shaped by the interplay of gender, migration status, ethnicity, and social class, as well as by the cumulative effects of trauma, stigma, and systemic discrimination [19].

The intersectional lens highlights overlapping layers of vulnerability and exclusion, while the trauma-informed perspective emphasises safety, empowerment, and the restoration of agency in all professional interactions. In this context, the term “integrated approach” refers to the coordinated and holistic provision of health, social, and legal support services, ensuring that care pathways are collaborative rather than fragmented. This framework guided both the design and interpretation of the study, offering a comprehensive understanding of the complex realities faced by migrant women victims of trafficking and sexual violence.

The present study is aimed at describing and understanding the experiences of health, social, and legal professionals in providing care to sub-Saharan migrant women, with particular attention to those who are victims of trafficking and/or sexual violence.

## 2. Materials and Methods

### 2.1. Study Design

A qualitative study was designed to explore and describe the experiences of health professionals, social workers, and legal practitioners who provide care to migrant women arriving irregularly in Spanish territory, with particular attention to victims of trafficking and/or sexual violence.

### 2.2. Participants and Setting

We used a purposive convenience sampling approach to intentionally recruit professionals from the health, legal, and social sectors who had direct experience supporting migrant and trafficked women in Almería. This strategy allowed the research team to access participants with diverse yet directly relevant perspectives on the phenomenon under study, ensuring representation across key professional profiles involved in victim care [20].

In this qualitative study, the participants were:

1. Health professionals specializing in medicine and nursing.
2. Legal professionals, specifically lawyers specialized in criminal law and immigration law.
3. Social professionals, staff from NGOs dedicated to assisting migrant women.

The sample consisted of three professional groups—health, legal, and social—selected according to specific inclusion and exclusion criteria. Physicians and nurses from the National Health Service, lawyers registered with expertise in criminal law and/or immigration law, and NGO members dedicated to migrant care were included, all with a minimum of six months of experience in assisting migrant women and who provided informed consent. Those who did not meet these requirements were excluded, and only adults were allowed to participate, thus ensuring the legal validity of consent. This criterion was established to ensure that participants had sufficient exposure to the realities and operational challenges of providing care to trafficked or sexually abused migrant women, while maintaining feasibility for recruiting active professionals across multiple institutions.

Given the high staff turnover and frequent short-term contracts within both healthcare and social service sectors in Andalusia, a six-month threshold was considered adequate to guarantee relevant experience and reflective insight without restricting participation to a very limited pool of experts.

The final sample was composed in Table 1, which provides an overview of the forty-seven professionals who participated in the study, grouped by sector, gender, and professional background. All participants worked in Almería and had more than six months of experience in their respective fields.

This study deliberately focused on professionals who work directly with trafficked women, as opposed to those assisting broader migrant or refugee populations. This purposive focus was methodologically consistent with the study’s objective: to obtain in-depth insights into the specific coordination, referral, and protection challenges that arise in cases of trafficking and sexual exploitation. By including only professionals with first-hand experience in this area, the research ensured conceptual precision and analytical depth, allowing a clearer identification of systemic barriers and cross-sector opportunities for improvement.

### 2.3. Data Collection

The identification and contact with participants were carried out through their respective reference institutions. Health professionals were recruited in hospital centers of the Andalusian Health Service located in the province of Almería and surrounding areas; legal professionals were identified through the Bar Association of Almería, accessing the roster of duty lawyers specialized in assisting migrants at points of mass entry; and social professionals were located through non-governmental organizations that met the inclusion criteria, distributed across the national territory.

Data collection was based on a guide of open-ended questions previously designed to address the core themes of the study, with the order and depth of the topics adapted according to each participant’s interaction. Interviews with health professionals were conducted in their workplaces, while those with legal and social professionals were carried out virtually through the platforms “Google Meet” and “WhatsApp,” facilitating compatibility with their schedules. The interview protocol for this three groups is presented in Table 2.

### 2.4. Data Management and Analysis

The interviews were conducted by two members of the research team (K.E.-M.-E.-F. and M.K.E.-A.E.), both nurses with extensive clinical and qualitative research experience in supporting migrant and trafficked women. Each interviewer transcribed and reviewed the interviews, storing the data in Word files for initial management and classification.

Transcriptions were independently reviewed by M.d.M.J.-L., a sociologist specializing in social intervention, and by M.J.R.V., a legal expert with professional experience in human rights and victim protection. This collaborative process ensured contextual accuracy, completeness, and consistency across transcripts, while the multidisciplinary exchange strengthened the credibility and transparency of the analysis.

An open coding process was applied to identify key themes and patterns and to establish the initial analytical categories. Thematic analysis was then conducted following the six-phase framework proposed by Braun and Clarke [21]. Transcripts were coded manually by two researchers (K.E.-M.-E.-F. and M.K.E.-A.E.), who independently identified meaningful units and developed initial codes. These codes were iteratively refined through group discussions until thematic saturation was achieved.

A detailed coding matrix in Excel was created to systematically trace excerpts, codes, and emerging themes across the 47 interviews. Although no specialized qualitative analysis software (e.g., NVivo or ATLAS.ti) was used, this structured Excel-based matrix ensured organization, traceability, and rigor throughout the analytic process. Intercoder reliability was enhanced through peer debriefing and double-coding of 20% of the transcripts, with discrepancies resolved through consensus among the researchers.

The coding process was further supported by iterative analytical meetings in which the research team collectively reviewed and refined codes, grouping them into broader categories and final themes. Constant comparison between interviews ensured that the resulting themes accurately reflected the diversity of participants’ experiences and perspectives.

Triangulation was achieved through collaboration among researchers from different disciplinary backgrounds (health, sociology, and law), enhancing both the validity and interpretive depth of the findings. Finally, the complete thematic structure was reviewed by an external qualitative research expert to confirm coherence, transparency, and consistency in the interpretation of results.

Specifically, K.E.-M.-E.-F., a nurse and legal expert experienced in human right and victim protection, and M.K.E.-A.E., a nurse with extensive clinical and qualitative research experience in the field of migrant and trafficked women’s health, conducted and transcribed the interviews. M.d.M.J.-L., an expert in qualitative research and social intervention, and M.J.R.V., a nurse with experience in qualitative research, reviewed and validated the transcripts. The entire research team contributed to the coding, thematic refinement, and interpretive analysis.

### 2.5. Ethical Criteria

The research protocol was approved by the Research Ethics Committee of the Department of Nursing, Physiotherapy and Medicine at the University of Almería (protocol number: EFM 225/2022). Participant confidentiality and anonymity were guaranteed by assigning each individual an identification code and providing a written informed consent form, which was signed prior to their inclusion in the study.

Data management was carried out in accordance with Organic Law 3/2018 of 5 December on the Protection of Personal Data and Guarantee of Digital Rights, Regulation (EU) 2016/679 of the European Parliament and of the Council of 27 April 2016 (GDPR), and Law 41/2002 of 14 November on Patient Autonomy and Rights and Obligations regarding Clinical Information and Documentation. All participants were informed of the study’s purpose and the voluntary nature of their participation, with informed consent obtained before the start and authorization requested for recording the interviews.

### 2.6. Rigor

To ensure rigor, the following criteria were applied: (a) Credibility: the data collection process was described in detail; (b) Triangulation: data were analyzed independently by the researchers and later compared in a joint discussion; (c) the researchers were health professionals and sociologists with extensive experience in care processes; (d) Transferability: detailed information was provided about the participants, study setting, context, and method; (e) Reliability and Confirmability: the researchers conducted the transcriptions, which were subsequently reviewed by other members of the research team.

The research team consisted of professionals from the fields of nursing, sociology, and law, each with extensive experience in healthcare provision, social work, and legal defence of migrant and trafficked women. Throughout the study, the researchers maintained a reflexive stance, regularly discussing how their professional backgrounds, values, and positionalities might influence data interpretation. These discussions fostered awareness of potential biases and ensured that analytical decisions were grounded in the participants’ voices rather than in researchers’ preconceptions. This reflexivity process enhanced transparency, credibility, and ethical integrity throughout the research.

## 3. Results

### 3.1. Theme 1: Overcoming Barriers: Challenges and Opportunities in Health Care for Migrant Women Victims of Trafficking and Sexual Violence 

This theme focuses on establishing and coordinating the group of professionals who provide care to a migrant woman victim of sexual assault, ensuring that their actions are homogeneous, coordinated, and respectful.

Before presenting the participants’ perspectives, it is important to contextualise the Spanish healthcare framework that shapes their experiences. The following introductory paragraph describes the institutional and regulatory context of healthcare provision for migrant women in Spain and is not derived directly from the interview data. This contextual note aims to clarify the structural environment in which the professionals’ narratives are situated.

Health care is directed toward women—regardless of their administrative status—who have experienced sexual violence or are suspected of having suffered it. For this reason, access to hospital facilities or gynecological care does not require a prior formal complaint.

#### 3.1.1. Subtheme 1.1 Comprehensive Approach in Health Care

It is essential to establish that all health care actions are organized within a multidisciplinary team composed of professionals from different specialties who share a common objective.

*“At the moment there are two teams in Almería city, each with six doctors and six nurses. We work on a rotating schedule, approximately one shift every six days, although we are also covering the Sorbas area due to the geographic dispersion there, in the Sorbas and Tabernas region. Each ambulance is staffed with a doctor, a nurse, and a technician.”*

(HS-2)

Spanish legislation itself guarantees that foreigners who are not residents in Spain are entitled to this right as a fundamental right to health protection, since they are holders of this right. One of the interviewees stated:

*“You know what happens? Migrants have the right to be treated effectively and even urgently, regardless of their administrative status...”*

(HS-17)

All necessary human and material resources must focus on respecting their personality, human dignity, and privacy, without discrimination based on race or ethnicity, gender or sexual orientation, disability, or any other personal or social circumstance. Therefore, it is essential that health services be culturally sensitive and designed to meet the unique cultural, social, and economic backgrounds of migrant women. One participant emphasized:

*“It is sad because many times you leave the patient just because their vital signs are more or less stable; the language barrier prevents you from really knowing what is wrong, and then you leave them in a place where you know it will be very difficult for anyone to check on them every four or five hours unless they themselves say they still feel unwell. So, it is a bit like leaving them adrift.”*

(HS-9)

#### 3.1.2. Subtheme 1.2 Beyond Illness: Promoting the Integral Health of Migrant Women Through Healthcare

The interviews reveal that the main difficulties and needs in the care of migrant women are linked to the coordination of competencies among different professionals. It is therefore essential to update and implement an effective protocol of action that overcomes both the inadequacy of custody facilities and the lack of adaptation of healthcare resources. As one participant pointed out:

*“There is no effective transfer of communication; many times when they call you from the modules, you arrive thinking you are going to find one thing and then you find ten, and you say: what an overflow, why don’t they say there are ten patients in this situation. So, we always end up running behind.”*

(HS-15)

Repeatedly, interviewees emphasized the need to increase professional experience, ensure better patient follow-up, and overcome language barriers, all of which directly affect the quality of care. One participant identified the lack of continuity of care as a particularly relevant issue:

*“One of the problems that I think we all see is patient follow-up, especially with babies. I remember many times the national police saying: but well, how am I supposed to be giving Ibuprofen and Augmentine that was prescribed at the maternal-infant hospital, who am I to be giving medication every eight hours?”*

(HS-8)

The language barrier emerges as a recurring obstacle, limiting the ability of professionals to communicate, understand needs, and clearly convey medical instructions:

*“The language barrier, although sometimes there is a translator and they do collaborate well, but it is not the same. And also, we are very guided by pathologies, but when a boat arrives, they tell you you’re going to see a boat, they’re not talking about pathologies, nor do you know any medical history; they’re talking about social situations, not pathologies, so you don’t know what you’re going into.”*

(HS-5)

Moreover, restrictions on access, deficiencies in clinical follow-up, and a lack of training in specific areas such as tropical medicine are highlighted. According to the professionals, improving this situation requires more training and a joint commitment from all the groups involved. One interviewee summarized it as follows:

*“It’s a problem; in my personal experience, they have come with their treatments and even with medical reports from Morocco and Algeria, and then it depends on the police inspector whether you explain it or not, whether they are given the medication or not. So, there are people who simply say no, and that’s it... It’s also a bit of a trend we have to criminalize the patient. If they are with the national police, it’s because they committed a crime...”*

(HS-14)

### 3.2. Theme 2: Rights Without Borders: Legal Assistance as a Tool for the Empowerment of Migrant Women

According to the Immigration Law, foreign nationals in Spain enjoy all the rights and freedoms guaranteed by the Spanish Constitution and by international treaties, being able to exercise their human rights on equal terms with Spanish citizens. Any act of discrimination must be avoided, understood as any action that, directly or indirectly, involves a distinction, exclusion, restriction, or preference against a foreigner based on race, color, ancestry or national or ethnic origin, as well as on religious beliefs and practices.

One of the fundamental rights protecting migrants is the right to effective judicial protection, which ensures that any person may initiate legal proceedings in order to obtain reparation for the violation of their rights. Another key right is the right to legal assistance. The Immigration Law stipulates that migrants within Spanish territory have the right to be assisted by lawyers in all proceedings in which they are involved, regardless of the jurisdiction, and always under the same conditions as Spanish citizens.

#### 3.2.1. Subtheme 2.1 Comprehensive Legal Protection for Migrant Women: A Human Rights Approach

The interviewees report that their way of organizing revolves around the duty shifts, which are assigned by the Bar Association. This institution manages both the general duty roster and the specialized roster, where all registered lawyers are included on an assistance list. One interviewee explained:

*“…the Bar Association notifies us that we are on the list within the top ten positions, and from that moment you have to stay alert for when the phone rings… and when they call you, you have to go to the police station to provide assistance…”*

(LS-3)

Foreign nationals present in Spain have the right to legal assistance in administrative procedures that may lead to their refusal of entry, return, or expulsion from Spanish territory, as well as in all procedures relating to international protection. They are also entitled to the assistance of an interpreter if they do not understand or speak the official language being used. This assistance will be free of charge if they lack sufficient financial resources, according to the criteria established in the regulations governing the right to free legal aid. One interviewee stated:

*“…assistance is provided through the duty roster; if a migrant contacts a private lawyer, it is usually because there is someone behind them willing to cover the fees… normally, the Bar Association keeps a list of all the professionals available for assistance and calls them in order… in summer the list usually moves faster, honestly, during the high season the duty roster advances quickly…”*

(LS-4)

With regard to legal assistance for victims of trafficking, it is essential that the lawyer carries out a confidential interview with the person to be assisted, regardless of whether the proceedings are criminal or administrative in nature. Several interviewees shared their experience, noting that:

*“…of course, when you get to the police station the papers are ready… you sign them… they stamp them and you leave… sometimes we do not even see the migrants to learn about their personal circumstances… and without knowing those circumstances, the administrative claims are not going to succeed…”*

(LS-10)

The most important part of legal assistance focuses on the identification of migrant women, safeguarding their integrity and rights, ensuring their access to protection through full legal assistance. The cornerstone lies in confidential communication, where their needs can be identified, including the possibility of filing applications for protection and asylum, within an effective framework of coordination between police officers, judges, and prosecutors. One participant emphasized:

*“…that is why I say things must change… we need to interview migrants individually and in private to gather all the information… whether they have families, if they are pregnant, if they are victims of trafficking… but if you are not allowed to do so, there is little you can achieve… we should also have full identification of migrants… many times birth data or even nationality are unknown… the entire system of assistance needs to change…”*

(LS-1)

#### 3.2.2. Subtheme 2.2 the Importance of Legal Assistance for Victims of Trafficking and Sexual Assault

The lawyer must conduct a confidential interview with the victim in order to understand her personal circumstances and the situation in which she finds herself.

*“…it is important to help people who arrive in such conditions; it is a key social and humanitarian task at an international level… we ourselves do not always appreciate the work we do… through our intervention, we can change the course of these people’s lives…”*

(LS-7)

It must also be taken into account that the victim may speak a language different from ours, in which case the intervention of an interpreter becomes necessary. At this point, it is important to highlight that not every foreigner speaks French or English; it is common for individuals to speak native languages for which it is difficult to find translation mechanisms. One interviewee stressed:

*“…personally, I would carry out the intervention in individual interview rooms and with an interpreter… but a specialized interpreter, not just anyone… because some do not speak well, and they harm us more than they help…”*

(LS-3)

It is advisable for the lawyer to request pre-constituted evidence for the victim as soon as the Court responsible for investigating the case is known. Likewise, the lawyer must request the application of the protected witness status, ensuring that the victim’s personal data are replaced in the proceedings with a numerical code. One female lawyer expressed:

*“…the whole system needs to change… the current system does not work… I would change the immigration law and the procedural law… so that the requirements for appearance to pursue legal remedies are not so harsh…”*

(LS-2)

### 3.3. Theme 3: Social Assistance as a Tool for the Integration of Migrant Women

The key to comprehensive care lies in providing emotional support and guidance when it comes to decision-making. In addition, it is essential to physically accompany migrant women to healthcare resources and police facilities, ensuring the exercise of the rights of victims of sexual violence while seeking to prevent their revictimization.

When it comes to care and protection for women in prostitution, victims of trafficking, or those in situations of clear exploitation, attention should focus on a multifocal and multisectoral approach, encompassing support through social centers and mobile units. Furthermore, holistic and comprehensive support is offered to all these migrant women, including psychological care, social assistance, employment guidance, referral services, and accompaniment.

These social professionals also focus on raising awareness among the general population by providing information about the phenomenon of trafficking and by training professionals who may come into contact with potential victims, thereby establishing themselves as a fundamental tool for prevention and early detection of victims.

#### 3.3.1. Subtheme 3.1 the Role of Social Assistance in the Lives of Migrant Women

The multicultural care provided by different NGOs strengthens migrant women who are victims of sexual assault or trafficking, breaking down social and cultural barriers that hinder their socio-labor integration.

*“We work in contexts of prostitution, in clubs, in apartments, in settlements, and we go to the places where women are in prostitution under the pretext of bringing preventive material, conducting HIV tests, and issuing health cards. Through all this, we begin to create a space to identify possible signs of human trafficking and to be able to support women with information and guidance.”*

(SS-5)

Social assistance can play a vital role in the lives of migrant women, offering a support network to help them face the challenges that may arise during the migration and integration process in a new country.

*“Our platform is basically one of awareness-raising and providing guidance to people who come to us with questions; we are essentially an assistance-oriented platform, in the sense of providing shelter and covering certain needs or deficiencies. That is what we mainly do. We are a small collective; most of us have other jobs, and it is very volunteer-driven. Therefore, we focus on this.”*

(SS-13)

Social assistance is carried out from a gender and intercultural perspective, focusing on the specific needs and characteristics of each migrant woman, developing tailored content that takes into account cultural and social diversity, while accompanying women to ensure their participation and integration. Other interviewees explained in their testimonies:

*“…it focuses on early diagnosis, awareness, and prevention of HIV and other STIs. Another focuses on the care of elderly people living in vulnerable neighborhoods in the province of Almería, not only in the capital but also in other municipalities, and another mainly on harm reduction for people in situations of prostitution and trafficking…”*

(SS-18)

Social assistance for migrant women victims of trafficking is essential to guarantee their recovery and effective reintegration into society. These women often face multiple barriers, including psychological trauma, social isolation, stigmatization, and health problems. In this regard, one participant stated:

*“When we already have a trafficking case, and we see that the woman is predisposed to report because she comes and tells the whole process, we write down the interview, send it to the Guardia Civil’s social liaison officer, and they summon her.”*

(SS-4)

Another participant explained that the way to reach trafficked women often begins with an informal conversation about another issue, and from there, the topic of trafficking is introduced, as follows:

*“…When we have had trafficking cases that were fairly evident, what we did was arrange to meet the woman here at the office. We usually approach it by discussing how she can regularize her administrative status. And through that explanation of how to regularize her situation, we start pulling the thread to see if there are signs of trafficking. This interview usually takes place in a less formal context, such as accompanying her somewhere by car, and when you have the opportunity to be alone with her, you start talking, and she begins to open up. Then you realize that the woman shows signs of trafficking, so we start monitoring her and explaining the situation, letting her know that she can file a complaint and that she could even obtain documentation, and so on.”*

(SS-11)

#### 3.3.2. Subtheme 3.2 Towards More Inclusive Social Assistance: Challenges and Opportunities for Migrant Women

All interviewees expressed the need to update protocols and have their own resources, since they receive little administrative support. This has a negative impact on the administrative situation of migrant women, as many do not have a health card and very limited resources. One professional interviewed reported the precarious conditions faced by migrants, explaining:

*“…They encounter many difficulties. The first is that they do not have the financial resources to cope with the situation they are in. They need social support, and many times they are afraid of going to social services or medical services precisely because they do not know whether they have that right or not, and they fear being detected, detained, or even sent back to their country of origin…”*

(SS-3)

Social assistance can provide emotional support, safe housing, medical care, counseling, and guidance for labor and social integration. One interviewee stressed the need to ensure prolonged access to healthcare, stating:

*“…I believe that within the public health system, both doctors and nurses have a duty to the community. Just as nursing and medical staff rotate through small towns— for example, I have worked as a nurse in several small towns covering four or five villages— I think mobile units for direct care should be set up in the areas where these people are located…”*

(SS-9)

These social practices focus on welcoming women by offering them protection and security, ensuring comprehensive care through programs that include social, psychological, and legal interventions needed to help them overcome the violence they have experienced. Participant SO-16 described:

*“…We also see that due to unemployment and the living conditions, besides physical problems, they develop many mental health issues—anxiety, depression, and even some develop schizophrenia. That is to say, there are countless physical and psychological health problems related to the conditions in which they live and their very precarious work situation. Imagine some people telling us they have been working for months without getting paid. And there is no solution…”*

(SS-16)

In addition, social assistance can play an important role in the early identification and prevention of trafficking, particularly considering issues such as:

*“Above all, the language barrier as well. We have seen 20 different diagnoses for the same illness and person, and that is due to inadequate translation…”*

(SS-17)

The main themes, subthemes, and corresponding units of meaning identified through thematic analysis are presented in Table 3.

## 4. Discussion

This study aimed to describe and understand the experiences of healthcare, social, and legal professionals regarding the care provided to sub-Saharan migrant women, particularly those who are victims of trafficking and/or sexual violence. The special protection granted by the Spanish legal system is based on the recognition that human trafficking disproportionately affects women for purposes of sexual exploitation, constituting a specific form of gender-based violence [22]. Victims of trafficking experience systematic violations of their human rights at multiple levels—social, economic, political, civil, cultural, and familial—whose interdependence often leads to processes of revictimization that amplify their vulnerability and the scope of the harm endured [23].

In line with these structural dynamics, the professionals’ accounts in this study consistently revealed that the fragmentation of health, social, and legal services further exacerbates the vulnerability of trafficked women. The lack of coordinated, intersectoral mechanisms limits continuity of care and reinforces the barriers already faced by these women in accessing protection and recovery pathways [24]. These findings underscore the urgent need for comprehensive and coordinated institutional responses that integrate healthcare, legal assistance, and social support under a shared human-rights framework [25].

Participants highlighted that healthcare for migrant women in Spain is formally grounded in the right to receive medical care regardless of their administrative status. In practice, this framework encompasses access to primary, specialized, obstetric, gynecological, and emergency services [26]. Migrant women with regular status—those holding a valid residence or work permit—enjoy the same healthcare rights as Spanish citizens, either through Social Security or the regional public health services [27].

By contrast, women in an irregular administrative situation, without a valid residence or work permit, are legally entitled only to emergency care in cases of serious illness or accident, care during pregnancy, childbirth and postpartum, and medical attention for minors [28]. Some autonomous communities have nevertheless developed specific programs extending primary and specialized care to this population [10].

The professionals interviewed emphasized that the right to health must be interpreted as universal and indivisible, meaning that a woman’s administrative status should never determine the quality, accessibility, or dignity of the healthcare she receives [1]. This perspective aligns with Spain’s human-rights-based approach to public health and underscores the need for institutional consistency between legal recognition and practical implementation of healthcare entitlements.

Migrant women may require a wide range of healthcare services similar to those of the general population [29]. However, their health demands are often intensified by the vulnerabilities associated with migration processes, including exposure to violence, precarious living conditions, and administrative instability [30]. These needs encompass access to primary and emergency care—such as preventive services, immunizations, reproductive and family planning care, and chronic disease management [31]—as well as obstetric and gynecological care, including prenatal, childbirth, and postpartum attention, and screening for cervical and breast cancer [32]. Depending on individual circumstances, many migrant women also require specialized medical attention, including diagnostic testing, chronic disease treatment, and both psychological and psychiatric support, alongside rehabilitation services [33].

Mental health emerged as a particularly critical area of concern, as migration experiences often generate post-traumatic stress, anxiety, depression, and grief, requiring sustained psychological and psychiatric care [34]. Within this context, some subgroups—such as pregnant women, victims of gender-based violence, women involved in prostitution or trafficking, and asylum seekers—demand highly specialized, trauma-informed healthcare interventions adapted to their specific vulnerabilities [35].

The range and intensity of these healthcare needs vary according to each woman’s health condition, administrative status, cultural background, and socioeconomic context [36]. Therefore, healthcare systems must adopt comprehensive, person-centered, and culturally sensitive approaches that integrate physical, psychological, and social dimensions of wellbeing. Providing holistic and respectful care is essential to address the complex realities of migrant women and to promote equitable access to health services across diverse populations [37].

All professionals—healthcare, legal, and social—agreed that violence against women, particularly sexual violence, constitutes a serious public health issue and a violation of fundamental human rights [38]. To acknowledge violence as a public health problem, it must be understood as a global phenomenon that perpetuates poor health outcomes throughout a woman’s life [39]. Therefore, policies and interventions must be grounded in scientific evidence and supported by research that addresses this issue from multiple perspectives [40].

Providing quality healthcare to migrant women who are victims of trafficking requires that health systems possess the necessary tools to ensure equitable, culturally competent, and trauma-informed care. Among these tools, cultural mediation stands out as an essential mechanism to bridge communication and trust gaps between migrants and healthcare providers, thereby improving service accessibility and patient understanding [41]. Many migrant women approach healthcare institutions with limited language skills and a pervasive fear of not being understood, which often inhibits full disclosure of their symptoms or experiences [42]. Despite these structural barriers, the professionals interviewed demonstrated strong adaptability, empathy, and commitment to delivering compassionate care within institutional constraints.

Therapeutic interventions should aim to restore dignity, rebuild self-esteem, and support the reconstruction of personal identity within the sociocultural frameworks that give meaning to each woman’s experience [43]. Healthcare professionals emphasized that well-designed interventions can strengthen the resilience of women who have suffered sexual assault [44]. Public health therefore plays a crucial role in a comprehensive approach to sexual violence, promoting protective factors for women’s physical and mental wellbeing. Studies on resilience in abused women reveal key mechanisms of adaptation, showing how survivors recover from trauma and rebuild successful lives and relationships [45].

In parallel, social support networks—comprising family members, friends, neighbors, and peers—play a fundamental role in recovery and reintegration [28]. These networks provide immediate assistance within shared social circles and act as informal but vital structures of emotional and material support [46]. Consequently, fostering intersectoral collaboration and ensuring trauma-informed and culturally sensitive practices emerge as essential priorities for both policy development and professional training. Social professionals described resilient women as demonstrating crucial attributes such as adaptability, coping ability, competence, and resistance to destruction—qualities that enable survival and reconstruction after extreme adversity [47].

The devastating consequences of sexual violence are well documented. Following rape, women frequently face isolation and severe social stigma, leading to profound physical and psychological harm, including genital mutilation and sexually transmitted infections such as HIV/AIDS [48]. Social and healthcare professionals reported that many sub-Saharan women require surgical reconstruction of genital or other organs damaged during assaults [49]. Both groups highlighted the most prevalent consequences of sexual violence: unwanted pregnancies, gynecological complications, sexually transmitted diseases, mental health disorders including depression and post-traumatic stress, suicidal behaviors, and social ostracism resulting from stigma within families or communities [50].

Social agents also underscored that the trafficking of women leads to their dehumanization, turning them into objects of exploitation within a consumerist system that ultimately deprives them of their rights and autonomy [51]. These experts associated the persistence of stress and vulnerability with factors such as discrimination, acculturative stress, and disruption of family structures during migration and separation processes [34].

From a legal perspective, professionals emphasized that sexual violence must not be understood as an isolated or individual act but rather as a social and structural phenomenon deeply embedded within cultural and institutional frameworks that sustain inequality and discrimination [52]. Beyond the individual harm inflicted, this form of violence has collective repercussions: it transmits messages of fear, subordination, and gender control that reinforce patriarchal norms and social hierarchies [48]. Moreover, professionals from all sectors recognized that exposure to such violence and its systemic consequences also affects them personally, posing a significant risk to their own mental health and emotional stability [53].

Taken together, these findings underscore that effective support for trafficked and sexually abused migrant women requires not only adequate resources but also a comprehensive, integrated, and human-rights-based response across all professional sectors. The trafficking of women for sexual exploitation attacks the very essence of personal freedom and dignity. Its eradication, therefore, must be assumed as a collective responsibility and a matter of State, demanding coordinated international action and robust cooperation among authorities and institutions. Only through public, intersectoral, and rights-based policies can societies guarantee justice, protection, and the restoration of dignity to victims of such violence [54].

## 5. Limitations

The results of this study expand the existing evidence on healthcare, social, and legal support for migrant women in vulnerable situations. The participants came from different professional fields, which allowed for the collection of diverse perspectives and added transferability to the findings. However, there are some limitations. First, the results are influenced by the sociocultural factors of the specific context in which the research was conducted. To contrast the findings, it would be advisable to carry out similar studies in other regions or countries. Second, although the study focuses on the views of professionals from different areas, future research could complement this by incorporating the direct perspective of migrant women themselves, thus enriching the understanding of the phenomenon.

While this study specifically focused on trafficked women, many of the systemic challenges identified—such as fragmented institutional coordination, insufficient cultural mediation, and inconsistencies in referral pathways—are likely to affect other vulnerable migrant populations as well. Nevertheless, caution should be exercised when generalising these findings. The legal recognition of trafficking victims, the specific forms of coercion and exploitation involved, and the procedural mechanisms available for protection create markedly different trajectories for trafficked women compared with refugees, asylum seekers, or other forced migrants.

Future research should therefore explore how the integrated framework proposed here could be adapted to other contexts of forced migration, and under what conditions such adaptations would remain effective.

## 6. Conclusions

The study shows that the care provided to migrant women victims of trafficking requires a comprehensive and multidisciplinary approach. Healthcare professionals highlight the need for continuous training and adequate resources to improve the quality of care. From the legal field, greater flexibility and capacity for intervention are demanded, emphasizing the importance of private interviews that allow for early detection and effective judicial protection. Meanwhile, social agents stress the need for close coordination between healthcare, legal, and law enforcement professionals in order to reduce the negative impact of trafficking on affected women.

Beyond identifying weaknesses across healthcare, social, and legal systems, this study emphasises the need for an integrated, trauma-informed, and intersectional framework that addresses the specific realities of trafficked and sexually abused migrant women. Strengthening cross-sector coordination, improving professional training in gender and cultural competence, and expanding specialised facilities should be prioritised by policymakers. Future efforts should also ensure that survivors’ voices are included in designing interventions that promote long-term empowerment and social reintegration.

## Figures and Tables

**Table 1:** Overview of participants’ professional and demographic characteristics.

Professional Sector	Participants	Gender (F/M)	Age Range (years)	City	Professional Background	Experience
Health sector (HS)	17	13 W 4 M	25–59	Almería	Medicine (10), Nursing (7)	More than 6 months
Legal sector (LS)	10	5 W 5 M	36–55	Almería	Criminal and Immigration Law	More than 6 months
Social sector (SS)	20	13 W 7 M	25–60	Almería	Social work, NGOs, community services	More than 6 months
Total Sample	47	31 W 16 M	25–60	Almería	—	More than 6 months

**Table 2:** Interview Protocol.

Phase	Type of Questions	Group of Health Professionals	Group of Legal Professionals	Group of Social Professionals
Presentation Phase	Study objective	We would like to learn about your experiences as a health professional in the care of migrants.	We would like to learn about your experiences as a lawyer providing assistance to migrant women.	We would like to learn about your experiences as a social worker providing assistance to migrant women.
	Ethical issues	Inform participants that their participation is voluntary and that they may withdraw at any time.	Inform participants that their participation is voluntary and that they may withdraw at any time.	Inform participants that their participation is voluntary and that they may withdraw at any time.
Initial Phase	Initial questions	Tell us about your experience as a health professional.	Tell us about your professional and personal experience in assisting migrants.	Tell us about your professional and personal experience in assisting migrants.
Development Phase	Questions on health care at the time of arrival	Tell us about your professional and personal experience in attending people arriving by boat.	How do you usually attend to and organize people arriving by boat?	What needs of migrants do you identify?
	Questions on health care during police custody	How do you coordinate with other professionals in providing assistance?	How do you usually gain access to migrants?	How is the detection of women victims of trafficking carried out?
	Questions on special needs	How do you provide care in cases with special needs?	What aspects do you think could be improved to facilitate your work?	What aspects do you think could be improved to facilitate your work?
Closing Phase	Final questions	What aspects do you think could be improved in your work? What difficulties do you encounter?	What difficulties do you encounter?	What difficulties do you encounter?

**Table 3:** Themes, subthemes, and units of meaning.

Theme	Subtheme	Units of Meaning
Overcoming Barriers: Challenges and Opportunities in Health Care for Migrant Women Victims of Trafficking and Sexual Violence	Comprehensive approach to health care	Fundamental right, Organization, Specialized care, Multidisciplinary team, Cultural awareness.
	Beyond illness: promoting the integral health of migrant women through health care	Breaking language barriers, Contingency plan, Improvements to be made, Health training for professionals, Institutional support.
Rights Without Borders: Legal Assistance as a Tool for Empowerment of Migrant Women	Comprehensive legal protection for migrant women: a human rights approach	Duty shifts, Assignment by the Bar Association, Legal aid, Specialized duty roster.
	The importance of legal assistance for victims of trafficking and sexual violence	Immediate assistance, Confidential interview, Language barrier, Administrative status, Protected witness.
Social Assistance as a Tool for the Integration of Migrant Women	The role of social assistance in the lives of migrant women:	Gender perspective, Cultural diversity, Cross-sectional care, Social and educational projects.
	Towards more inclusive social assistance: challenges and opportunities for migrant women:	Updating protocols, Own resources, Emotional support, Guidance, Regularization.

## Data Availability

The data presented in this study are available on request from the corresponding author. The data are not publicly available due to privacy concerns and ethical restrictions, as they derive from interviews with healthcare, legal, and social professionals working with migrant women victims of human trafficking. Access to the data is subject to the ethical approval obtained (EFM 225/2022, University of Almería).
